# Thyrotoxic Periodic Paralysis With Features of Andersen-Tawil Syndrome: A Case Report and Literature Review

**DOI:** 10.7759/cureus.8169

**Published:** 2020-05-17

**Authors:** Beshoy Iskander, Bilal Haider Malik, Ivan Cancarevic

**Affiliations:** 1 Internal Medicine, California Institute of Behavioral Neurosciences and Psychology, Fairfield, USA

**Keywords:** thyrotoxic periodic paralysis, andersen-tawil syndrome, long qt syndrome, long qt 7, periodic paralysis, hypokalemic periodic paralysis, thyrotoxicosis, long qt., mandibular hypoplasis, paralysis

## Abstract

Thyrotoxic periodic paralysis (TPP) is a rare manifestation of hyperthyroidism. The pathophysiology of hyperthyroidism causing periodic paralysis involves the Na+/K+ ATPase and potassium channels. We present a case of a 30-year-old male who presented to the ED with acute onset of upper and lower limb weakness. The patient was found to have bilateral weakness in the upper and lower limbs, orbital hypertelorism, and mandibular hypoplasia. He was also found to have hypokalemia, low thyroid-stimulating hormone (TSH), elevated thyroid peroxidase antibody, and elevated thyroid-stimulating immunoglobulins. The patient’s EKG was remarkable for a prolonged QTc interval. The patient regained his muscle strength after potassium replacement in less than 24 hours. He was started on methimazole and potassium supplements. Our case is unique because it shows the possibility of the presence of Andersen-Tawil syndrome (ATS) (long QT syndrome 7), diagnosed by the presence of periodic paralysis, long QT, and dysmorphic facial features with TPP. In conclusion, thyrotoxicosis can trigger ATS; also the two syndromes can co-exist owing to the similarity in their pathophysiology.

## Introduction

Thyrotoxic periodic paralysis (TPP) is one of the rare manifestations of hyperthyroidism [1,2]. TPP is more common in the Asian population; fewer cases were reported on the Caucasian and black population [1]. TPP is characterized by recurrent attacks of reversible muscle weakness and hypokalemia [3]. Studies support that hyperthyroidism, hyperinsulinemia, and androgen stimulate the Na+/K+ ATPase activity. Increased thyroid hormone inhibits K efflux channels leading to trapping K+ inside the cells and subsequent alteration in repolarization in skeletal muscles [2]. 

Andersen-Tawil Syndrome (ATS) is a primary periodic paralysis that can present as an autosomal dominant or a sporadic disorder [4,5]. Some cases of ATS are caused by a mutation in KCNJ2 gene coding for inward rectifier potassium channel, which stabilizes the resting membrane potential in skeletal and cardiac myocytes [4]. Unlike TPP, ATS tends to affect multiple sites, including cardiac and skeletal cells leading to its clinical presentation of a triad of hypokalemia, prolonged QTc, and facial and skeletal dysmorphism (low-set ears, mandibular hypoplasia, orbital hypertelorism) [4,5].

## Case presentation

**Figure 1:**
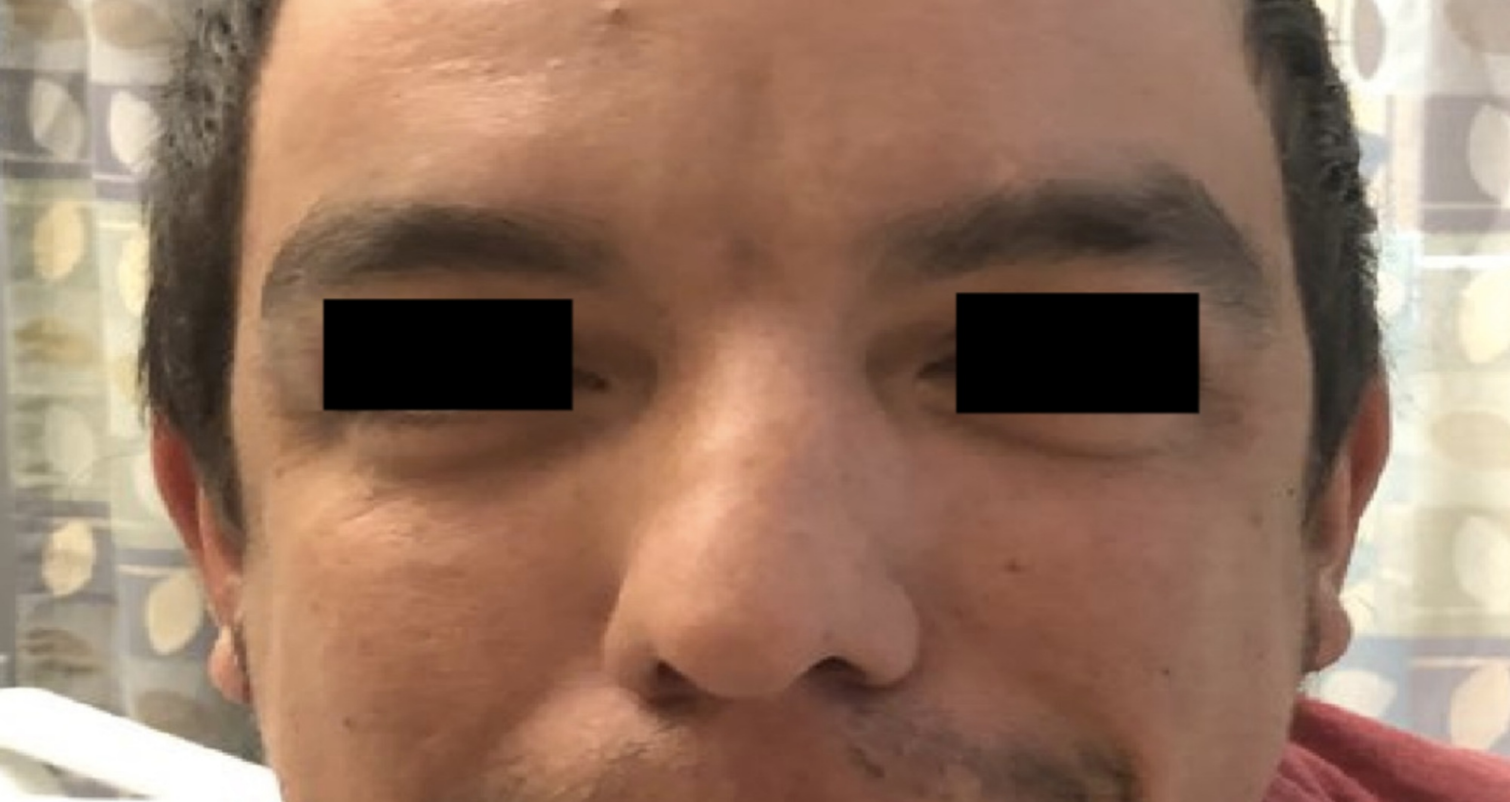
Orbital hypertelorism

**Figure 2:**
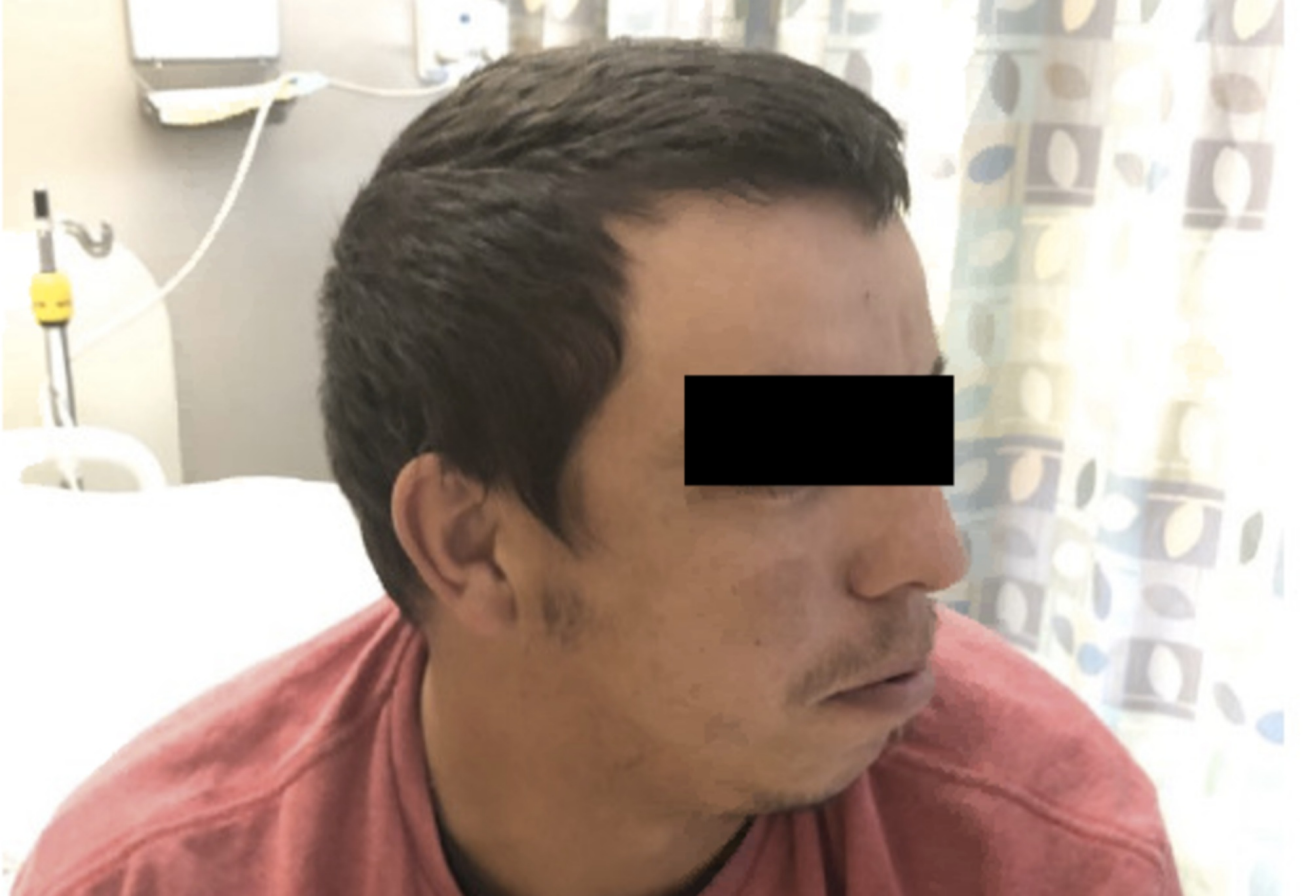
Mandibular hypoplasia

**Figure 3:**
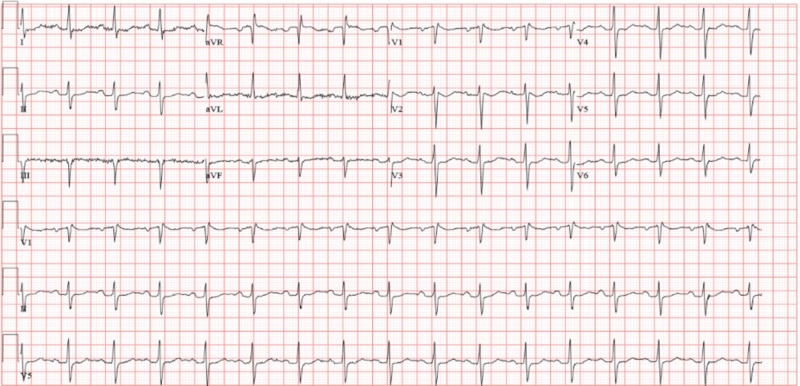
Prolonged QT (QU) interval identified in lead II using Bazzett's correction formula

A 30-year-old Caucasian male with a past medical history of periodic paralysis, taking no home medications, presented to the ED with lower extremity and upper extremity weakness after drinking about ten cans of soda and energy drinks. A review of systems was unremarkable for arrhythmias in the past. Family history was not significant for a similar disease process and was only positive for hypertension in his mother. Social history was not remarkable. On presentation, the patient was tachycardic at 105 beats/minute. Physical examination was notable for orbital hypertelorism (Figure 1), mandibular hypoplasia (Figure 2), 3/5 strength in the upper extremities bilaterally, 2/5 in the lower extremities bilaterally, +1 reflexes in the bilateral upper and lower extremities, normal overall muscle tone, and no sensory deficits. The patient’s relevant laboratory date is displayed in Table 1. The patient’s initial EKG was remarkable for a manually calculated QTc interval using the Bazett technique of 537 msec (Figure 3). The patient was then given a total of 140 meq KCL within 48 hours of his hospital stay with regaining his full motor strength and was discharged home with methimazole 5 mg three times daily and KCL 20 meq daily. 

**Table 1:** Laboratory values TSH, thyroid-stimulating hormone; free T3, free triiodothyronine; free T4, free thyroxine

Laboratory investigation	Patient’s value	Reference value
Potassium	2.2 mmol/L	3.5-5 mmol/L
Magnesium	1.6 mg/dL	1.6-2.6 mg/dL
TSH	<0.010 uIU/mL	0.270-4.700 uIU/mL
Free T3	9.4 pg/mL	2-4.4 pg/mL
Free T4	3.04 ng/dL	0.93-1.70 ng/dL
Thyroid-stimulating immunoglobulin	16.80 IU/L	< = 0.54 IU/L
Thyroid peroxidase antibody	299.8 IU/L	0.0-9.0 IU/L
Spot urine calcium	12.0 mg/dl	Not identified
Spot urine phosphorus	<4 mg/dl	Not identified
Urine potassium to creatinine ration	2.18	Not identified

## Discussion

In TPP, insulin is induced by two ways: (1) increased thyroid hormone (T4) and (2) carbohydrate load [1,2]. Insulin then influences the Na+/K+ ATPase, altering the membrane permeability, causing alteration K+ levels [1]. Elevated thyroid hormone will lead as well to increase the sensitivity of N+/K+ ATPase to beta-adrenergic stimulation [1,2]. The increase in Na+/K+ ATPase activity is often compensated by an appropriate K+ efflux leading to a balance in the K+ in the extracellular fluid [2]. That leads the conclusion that a defect in the Na+/K+ ATPase activity is not only responsible for hypokalemia. However, insulin and catecholamines decrease the activity of K+ efflux leading to fewer K+ in the extracellular fluid [2]. Few studies have reported a mutation in the Kir2.6 encoding gene, a skeletal-muscle specific Kir channel in a particular population, and predispose to acute paralysis [6].

ATS can be either an autosomal dominant disease or a sporadic disorder that is characterized by a triad of (1) periodic paralysis, (2) long QT/ventricular arrhythmias, and (3) dysmorphic facial features [4,5]. ATS is characterized by a mutation in the KCNJ2 gene on chromosome 17Q23 that codes for the potassium channel Kir2.1 [4,5]. Our patient exhibited all three features. QT interval was prolonged on presentation. However, a review of systems was unremarkable for any arrhythmias in the past. Measuring QTc interval has been a challenging part of our case, there is no agreement whether the U wave should be included in the measurement of the QT interval generally; however, in long QT syndromes, it is a usual part of the practice to include the U wave (QU interval) since ventricular repolarization ends by the end of the U wave [4,5]. The QTc was calculated using leads II and V5 as proposed by most of the experts using Bazzett’s correction formula. The tangent method was used to determine the end of the T wave and was 537 msec [7]. Dysmorphic facial features were also noted on physical examination by the presence of orbital hypertelorism and mandibular hypoplasia.

Both TPP and ATS involve the regulation of potassium across the cell membranes in their pathophysiology [1-5]. Alteration in the gene coding for Kir2.6 (subfamily of potassium inward rectifying channels) was found in 33% of TPP cases [6,8]. The similarities in the structure and the family of the potassium inward rectifying channels in both diseases [6,8] raise the possibility of the two disorders co-existence, although the involvement of different genes has been reported. Diaz-Manera et al. have reported exacerbation of symptoms due to thyrotoxicosis in a patient with an already established ATS diagnosis [9]. Our case might represent an exacerbation caused by thyrotoxicosis from Grave’s disease or a co-existence of both disorders. 

Maintaining a euthyroid state is considered the cornerstone in preventing further paralysis attacks in TPP [1,3]. Avoiding a high carbohydrate diet, alcohol intake, and extreme exertion are necessary, especially before achieving the euthyroid state [1,3]. The use of non-selective beta-blockers, especially propranolol, has also been recommended by many authors [1]. Although replacing potassium during acute attacks of TPP has already established its importance, the use of potassium between the attacks for prophylaxis is not under the agreement [1]. Some authors suggested that there is no role for potassium supplements in preventing further attacks, and other authors suggested high dose supplements [1]. We decided to start our patient on low dose potassium supplements and recommended close follow-up. 

## Conclusions

ATS is diagnosed based on a triad of (1) periodic paralysis (hypo or hyperkalemia), (2) dysmorphic features, and (3) cardiac abnormalities (prolonged QTc interval, prominent U wave, and ventricular arrhythmias). Our case is unique because it shows the possibility of co-existence or triggering-relationship between TPP and ATS. This fact will suggest overlapping between different types of channelopathies.
